# Transplantation of MiR-28-5p-Modified BMSCs Promotes Functional Recovery After Spinal Cord Injury

**DOI:** 10.1007/s12035-023-03702-3

**Published:** 2023-10-21

**Authors:** Zhen Li, Haitao Su, Guandai Lin, Kai Wang, Yongming Huang, Yaqian Wen, Dan Luo, Yu Hou, Xuewei Cao, Jiaxian Weng, Dingkun Lin, Le Wang, Xing Li

**Affiliations:** 1 State Key Laboratory of Dampness, Syndrome of Chinese Medicine, Department of Orthopedic Surgery, The Second Affiliated Hospital of Guangzhou University of Chinese Medicine, Guangzhou, 510120 Guangdong China; 2 The Second Clinical College of Guangzhou, University of Chinese Medicine, Guangzhou, 510120 Guangdong China; 3 Guangzhou University of Chinese Medicine, Guangzhou, 510405 Guangdong China; 4 Lingnan Medical Research Center of Guangzhou University of Chinese Medicine, Guangzhou, 510405 Guangdong China; 5 Department of Spine Surgery, the First Affiliated Hospital of Sun Yat-Sen University; Guangdong Provincial Key Laboratory of Orthopedics and Traumatology, Guangzhou, 510080 Guangdong China

**Keywords:** Bone marrow mesenchymal stem cells, Neuronal differentiation, Spinal cord injury, miR-28-5p, Notch signaling pathway

## Abstract

Traumatic spinal cord injury (TSCI) is a prevalent central nervous system condition that imposes a significant burden on both families and society, affecting more than 2 million people worldwide. Recently, there has been increasing interest in bone marrow mesenchymal stem cell (BMSC) transplantation as a promising treatment for spinal cord injury (SCI) due to their accessibility and low immunogenicity. However, the mere transplantation of BMSCs has limited capacity to directly participate in the repair of host spinal cord nerve function. MiR-28-5p, identified as a key differentially expressed miRNA in spinal cord ischemia–reperfusion injury, exhibits differential expression and regulation in various neurological diseases. Nevertheless, its involvement in this process and its specific regulatory mechanisms in SCI remain unclear. Therefore, this study aimed to investigate the potential mechanisms through which miR-28-5p promotes the neuronal differentiation of BMSCs both in vivo and in vitro. Our results indicate that miR-28-5p may directly target Notch1, thereby facilitating the neuronal differentiation of BMSCs in vitro*.* Furthermore, the transplantation of lentivirus-mediated miR-28-5p-overexpressed BMSCs into SCI rats effectively improved footprint tests and Basso, Beattie, and Bresnahan (BBB) scores, ameliorated histological morphology (hematoxylin–eosin [HE] and Nissl staining), promoted axonal regeneration (MAP2 and growth-associated protein 43 [GAP43]), and facilitated axonal remyelination (myelin basic protein [MBP]). These findings may suggest that miR-28-5p-modified BMSCs could serve as a therapeutic target to enhance the behavioral and neurological recovery of SCI rats.

## Introduction

Traumatic spinal cord injury (SCI) is a prevalent condition of the central nervous system that often leads to dysfunctions in the spinal cord below the level of injury, affecting movement, sensation, and reflexes. Over 2 million individuals suffer from SCI worldwide, with approximately 180,000 new cases reported annually, resulting in high rates of disability and mortality, placing a significant burden on both families and society [1, 2]. Despite advancements in therapeutic approaches over recent decades, obstacles such as axonal regeneration and neurogenesis still hinder the recovery of neural function after SCI [3, 4].

In recent years, cell transplantation has emerged as a highly promising treatment for SCI [3, 5]. Among the various options, bone marrow mesenchymal stem cells (BMSCs) have demonstrated superiority in clinical research owing to their availability and low immunogenicity [6, 7]. Previous studies have shown that BMSC transplantation can promote nerve repair [8–10]. However, the simple transplantation of BMSCs to differentiate into nerve cells and directly contribute to the repair of host spinal cord nerve function has limited efficacy [11]. Therefore, it is crucial to explore mechanisms that enhance the neuronal differentiation of BMSCs and improve the survival rate of neuronal cells post-differentiation.

Furthermore, miRNAs are endogenous single-stranded non-coding small (17–24 nt) RNAs that induce mRNA degradation or inhibit translation [12]. Some miRNAs play regulatory roles in mammalian neuronal differentiation processes including inflammation, regeneration, apoptosis, and demyelination [13, 14]. Previous studies have revealed differential expression and regulation of miR-28-5p in various neurological diseases [15–17]. Additionally, miR-28-5p has been found to alleviate neuropathic pain in rats with chronic sciatic nerve injury by downregulating Zeb1 serum extracellular vesicle-derived miR-28-5p, which shows potential as a biomarker for Parkinson’s disease [15]. Moreover, miR-28-5p has been identified as a key differentially expressed miRNA in spinal cord ischemia–reperfusion injury and may serve as a potential intervention target [16]. Thus, miRNAs play a crucial role in the neuronal differentiation of BMSCs [18]. However, the involvement of miR-28-5p in this process and its specific regulatory mechanisms remain unclear.

Consequently, in this study, we examined the effects and underlying mechanisms of miR-28-5p on the neuronal differentiation of BMSCs in vitro. Furthermore, we investigated the functional recovery from SCI in vivo by transplanting miR-28-5p-modified BMSCs. Our findings were aimed to enhance the efficiency of neuronal differentiation and the post-differentiation survival rate of BMSCs, offering a promising therapeutic target for SCI repair.

## Materials and Methods

### Isolation, Culture, and Neuronal Differentiation of BMSCs

This study was approved by the Animal Ethics Committee of Guangzhou University of Chinese Medicine and adhered to all animal ethics standards. BMSCs were obtained from embryonic 2-week-old male Sprague–Dawley (SD) rats at the Experimental Animal Center, Guangzhou University of Chinese Medicine, Guangzhou, China, as previously described [19]. The procedure was performed under sterile conditions by separating the femur and tibia of both lower extremities, exposing the marrow cavity, and flushing the bones with phosphate-buffered saline (PBS) until they turned pale. Subsequently, a cell suspension was obtained and centrifuged at 1500 rpm for 5 min. BMSCs were then resuspended in MEM Alpha basic medium (1X; Gibco, Life Technologies, USA) supplemented with 10% fetal bovine serum (FBS; Gibco, Life Technologies, USA), 100 U/mL of penicillin, and 100 mg/mL of streptomycin (Gibco, Life Technologies, USA). The cells were cultured in α-MEM under conditions of 37 °C, 5% CO_2_, and saturated humidity. The medium was changed every 2 days, discarding non-adherent cells.

To induce neuronal differentiation, BMSCs were digested with 0.25% trypsin (Gibco) and then seeded into 12-well tissue-culture plates at a density of 2 × 10^4^ cells per well. Subsequently, the cells were grown for 12 days in DMEM-F12 (1:1) basic medium (1X; Gibco) supplemented with 1% penicillin and streptomycin (Gibco), 1% N2 Supplement CTS™ (100X; Gibco), 2% B27™ Supplement (50X; Gibco), 0.5% FBS (Gibco), 20 ng/mL of brain-derived neurotrophic factor (BDNF; PeproTech, Rocky Hill, NJ, USA), 10 ng/mL of epidermal growth factor (EGF; PeproTech), 10 ng/mL of basic fibroblast growth factor (bFGF; PeproTech), and 1% l-glutamine (Gibco).

### Luciferase Reporter Assay

The miR-28-5p target gene was predicted using the online software TargetScan 7.1 (http://www.targetscan.org/vert_71/). To obtain the Notch1-WT (wild type) and Notch1-MUT (mutant) plasmids, DNA fragments containing the normal and mutant binding sites of miR-28-5p in the 3′-untranslated region (3′-UTR) of Notch1 were inserted into the luciferase reporter gene plasmid. Subsequently, miR-28-5p mimics and plasmids were co-transfected into 293 T cells. Additionally, 293 T cells were co-transfected with negative control (NC) oligonucleotides and plasmids as controls. Finally, the luciferase activity was determined using the luciferase assay.

### Transfection of BMSCs

BMSCs were resuspended into single-cell suspensions 2 days prior to transfection and then seeded into a six-well plate at a density of 2 × 10^5^ cells per well. The BMSCs were transfected with 50 nM miR-28-5p mimics, 100 nM miR-28-5p inhibitor, or negative control (RIBO Biotechnology Company, Guangzhou, China) using the Lipofectamine 2000 reagent when the cells reached a confluence of 70–80%. The effects of cell transfection were analyzed by quantitative reverse transcription polymerase chain reaction (qRT-PCR) 48 h post-transfection.

### Construction of Recombinant Lentiviral Vectors

The sequence of miR-28-5p was retrieved from NCBI to facilitate the design of amplification primers. Rat DNA served as the template for PCR amplification, allowing recovery and amplification of the miR-28-5p DNA fragment through agarose gel electrophoresis. Subsequently, the resulting miR-28-5p DNA fragment was ligated to a lentiviral vector containing green fluorescent protein (GFP). The positive clone plasmid was then transformed and screened, followed by verification through sequencing. Finally, it was named miR-28-5p-GFP.

Next, 293 T cells were resuscitated and expanded in culture. Co-transfection of these cells involved the miR-28-5p-GFP plasmid along with two auxiliary packaging plasmids using Lipofectamine 2000. After 48 h of incubation, the supernatant from the 293 T cells was collected, filtered (0.45 µm), and subsequently ultracentrifuged at 4 °C for 2 h to concentrate the virus. The supernatant was discarded, and the preservation solution was added. This procedure resulted in the production of the LV-miR-28-5p-GFP lentivirus.

Subsequently, the lentivirus was serially diluted and employed to infect 293 T cells. Four days later, the number of fluorescent cells was determined, enabling the calculation of the virus titer. Following the determination of the optimal multiplicity of infection (MOI), the lentivirus was utilized to infect the target cells.

### Establishment of SCI Model and Cells Transplantation

Forty-eight male-specific pathogen-free (SPF) Sprague–Dawley (SD) rats weighing 200–250 g were procured from the Experimental Animal Center of Guangzhou University of Chinese Medicine. The rats were housed in cages with a 12-h light/dark cycle and allowed to acclimate for approximately 1 week.

The SCI model was created following the Allen method [10, 20, 21]. After anesthetizing the rats, their dorsal hair was shaved. Under sterile conditions, a midline incision of 2 cm was made at the T10 level. The muscle was then separated from the pedicle, and the T10 spinous process was stripped. Subsequently, a laminectomy was performed at the same level, followed by creating a longitudinal midline incision of approximately 0.5 cm to expose the T10 spinal cord, which was struck through a pneumatic vertical device to simulate SCI. The impact parameters were set as follows: impact speed of 1.2 m/s, impact depth of 1.0 mm, and rest time of 85 ms. After suturing the wound, the body temperature of the rats was maintained at 37.0 ± 0.5 °C. After modeling, the rats’ bladder was manually compressed twice daily to induce urination. In the sham-operated group, each rat underwent laminectomy without contusion injury.

The rats were randomly divided into four groups (*n* = 12): Sham, TSCI, Lv-Con-treated BMSCs, and Lv-miR-28-5p-treated BMSCs. One day after successful establishment of the SCI model, the rats were injected with either sterile saline or BMSCs via the tail vein. In the Sham and TSCI groups, the rats received a 1 mL injection of sterile saline. The Lv-Con group was administered a single-cell suspension (2 × 10^6^ cells/mL) of BMSCs transfected with the negative control lentiviral vector, also in a 1 mL volume. The Lv-miR-28-5p group was injected with 1 mL of a single-cell suspension (2 × 10^6^ cells/mL) of BMSCs transfected with miR-28-5p-overexpressing lentiviral vector.

### Behavior Assessment

The Basso, Beattie, and Bresnahan (BBB) locomotor test was previously utilized to assess hindlimb motor function in a lumbar spine injury model [22]. A score of 0 indicates the absence of significant movement in the hind limbs, while a score of 21 signifies normal hind limb movement. In our study, all animals underwent behavioral assessments at 24 h after spinal cord contusion, as well as on days 7, 14, and 21 following BMSC transplantation. The BBB scoring procedure was carried out by observers who were familiar with the scoring rules but were blinded to the experimental content.

### Histological Assessment

Twenty-one days after transplantation of BMSCs, rats were euthanized, and the spinal cord tissue was removed by transcardial perfusion with 0.9% saline and fixed using 4% paraformaldehyde (PFA). Subsequently, dehydration was carried out using graded ethanol concentrations (70%, 80%, 95%, and 100%), followed by embedding in paraffin. The resulting wax blocks were trimmed and sectioned at a thickness of 5 µm using a microtome. These sections were then subjected to overnight baking at 37 °C and subsequently stained with hematoxylin–eosin (HE) and Nissl stains. For the assessment of the inflammatory cavity area surrounding the lesions, three animal samples from each group were selected for HE staining [23]. Furthermore, Nissl staining was performed on three animal samples from each group to evaluate neuron survival. The number of positively stained cells was quantified in a blinded manner.

### Immunofluorescent Staining of Cells and Tissue Sections

In the immunofluorescence assay, neural-differentiated BMSCs were fixed with 4% PFA for 30 min and permeabilized using 0.3% Triton X-100 at room temperature (RT) for the same duration. Tissue sections underwent xylene and graded alcohol treatments, followed by antigen retrieval using 0.01 M citric acid (pH 6.0) at 95 °C for 10 min. The initial blocking step involved incubating the samples with 10% FBS (Gibco, Life Technologies, USA) for 1 h at RT. Subsequently, tissues or cells were subjected to overnight incubation at 4 °C with primary antibodies, including microtubule-associated protein 2 (MAP2) at a dilution of 1:200 (Boster Biological Engineering Co.), neuron-specific enolase (NSE) at a dilution of 1:200 (Millipore), β3-tubulin at a dilution of 1:200 (Boster Biological Engineering Co.), neurofilament 200 (NF-200) at a dilution of 1:400 (CST), Notch1 at a dilution of 1:200 (Abcam), glial fibrillary acidic protein (GFAP) at a dilution of 1:600 (Boster Biological Engineering Co.), growth-associated protein 43 (GAP43) at a dilution of 1:200 (NOVUS), and myelin basic protein (MBP) at a dilution of 1:200 (Boster Biological Engineering Co.). The following day, secondary antibodies conjugated with Alexa Fluor® fluorochrome (at a dilution of 1:300, Invitrogen) were utilized to detect the respective primary antibodies, while the cell nuclei were counterstained with DAPI. Finally, fluorescence images were observed using an Olympus IX73 fluorescence microscope.

### qRT-PCR

The qRT-PCR was performed to evaluate the expression of miR-28-5p and Notch1 at day 7, along with neuronal markers (MAP2, NSE, β3-tubulin, and NF200), in neural-differentiated BMSCs and spinal cord tissues 21 days after the transplantation of miR-28-5p-modified BMSCs. To extract total RNA from neural-differentiated BMSCs or spinal cord segments containing the injury epicenter, we utilized an RNAiso Plus Kit (Takara Biotechnology). Subsequently, reverse transcription was performed using the PrimeScriptRT kit (Takara Biotechnology). For qPCR, the SYBR® Green Premix *Pro Taq* HS qPCR kit II (Accurate Biotechnology, Guangzhou, China) was employed. The PCR reaction mixture consisted of 2 µL of cDNA solution, 10 µL of 2xSYBR® Green *Pro Taq* HS Premix II, 0.8 µL of PCR forward primer, 0.8 µL of reverse primer (both at 10 µM), and RNase-free water to make a final volume of 20 µL. The downstream primers for miR-28-5p were created using a universal downstream primer kit. A two-step PCR reaction program was implemented with the following conditions: Step 1 involved pre-denaturation at 95 °C for 30 s, followed by Step 2 consisting of 40 cycles of denaturation at 95 °C for 5 s, and annealing and extension at 60 °C for 20 s. U6 served as the internal reference. The 2^−ΔΔCt^ method was utilized to evaluate the expression of miR-28-5p, Notch1, and the neuronal markers. The specific primer sequences are provided in Table 1.

**Table 1:** Primer sequences for qRT-PCR

Gene	Primer sequence
miR-28-5p	5′-CGCAAGGAGCTCACAGTCTATTGAG-3′
U6	F:5′-GCTTCGGCAGCACATATACTAAAAT-3′
	R:5′-CGCTTCACGAATTTGCGTGTCAT-3′
Notch1	F:5′-GCCAGCAAGAAGAAGCGGAGAG-3′
	R:5′-CCACTCGTTCTGATTGTCGTCCATC-3′
MAP2	F:5′-CCACCATCGCCAGCATCAGAAC-3′
	R:5′-CTCCATTTCCTCAGCCATCACACC-3′
NSE	F: 5′-ACCACATCAACAGCACCATCGC-3′
	R:5′-GCATCAGGTTGTCCAGCTTCTCC-3′
β3-tubulin	F:5′-CATGAAGGAGGTGGATGAGCAGATG-3′
	R:5′-GTTGCCGATGAAGGTGGACGAC-3′
NF200	F:5′-TGGACATTGAGATTGCCGCTTACAG-3′
	R:5′-AGAGAGAAGGGACTCGGACCAAAG-3′
GAPDH	F:5′-ACCCAGAAGACTGTGGATGG-3′
	R:5′-GAGGCAGGGATGATGTTCTG-3′

*F* forward, *R* reverse

### Western Blotting

The protein levels in neural-differentiated BMSCs and spinal cord tissues, 21 days after the transplantation of miR-28-5p-modified BMSCs, were detected using Western blots. The total protein from each sample was extracted with radio immunoprecipitation assay (RIPA) lysis buffer (Gibco, Grand Island, NY, USA). Initially, the RIPA lysis buffer was added to the tissues and then mixed with an ultrasonic pulverizer, followed by two centrifugations at 12,000 g for 10 min at 4 °C. The supernatant was collected, and the protein concentration was determined using a bicinchoninic acid (BCA) kit (Bio-Rad Laboratories, CA, USA). Subsequently, a 20 μg/10 μL protein solution was prepared using a loading buffer and denatured at 99 °C for 10 min. The protein solution was stored at − 20 °C. Next, 20 μg of total protein from each group was separated on a 10% sodium dodecyl sulfate polyacrylamide gel electrophoresis (SDS-PAGE) gel and transferred onto polyvinylidene difluoride (PVDF) membranes. The membranes were then blocked with 5% milk and incubated overnight at 4 °C with primary antibodies, including MAP2 (1:1000; Boster Biological Engineering Co.), NSE (1:1000; Abcam, England), β3-tubulin (1:1000; CST), NF-200 (1:1000; Boster Biological Engineering Co.), Notch-1 (1:1000; Abcam, England), and GAPDH (1:5000; Thermo Fisher). The membranes were then washed with Tris buffer solution (TBST) and incubated with the secondary antibody (1:1000) for 90 min at room temperature (RT). Protein visualization was performed using a ChemiDoc™ MP imaging system (Bio-Rad), and the relative intensities of each band were determined using Image J software (National Institutes of Health, Bethesda, MD). GAPDH served as the internal reference.

### Statistical Analyses

SPSS 20.0 (SPSS, Chicago, IL, USA) was applied for statistical analyses. The results are presented as mean ± standard deviation (SD). One-way analysis of variance (ANOVA) was employed to compare multiple groups, whereas unpaired Student’s *t*-tests were used to analyze differences between two groups. A significance level of *p* < 0.05 was considered statistically significant.

## Results

### BMSCs Successfully Promoted Neuronal Differentiation

**Fig. 1:**
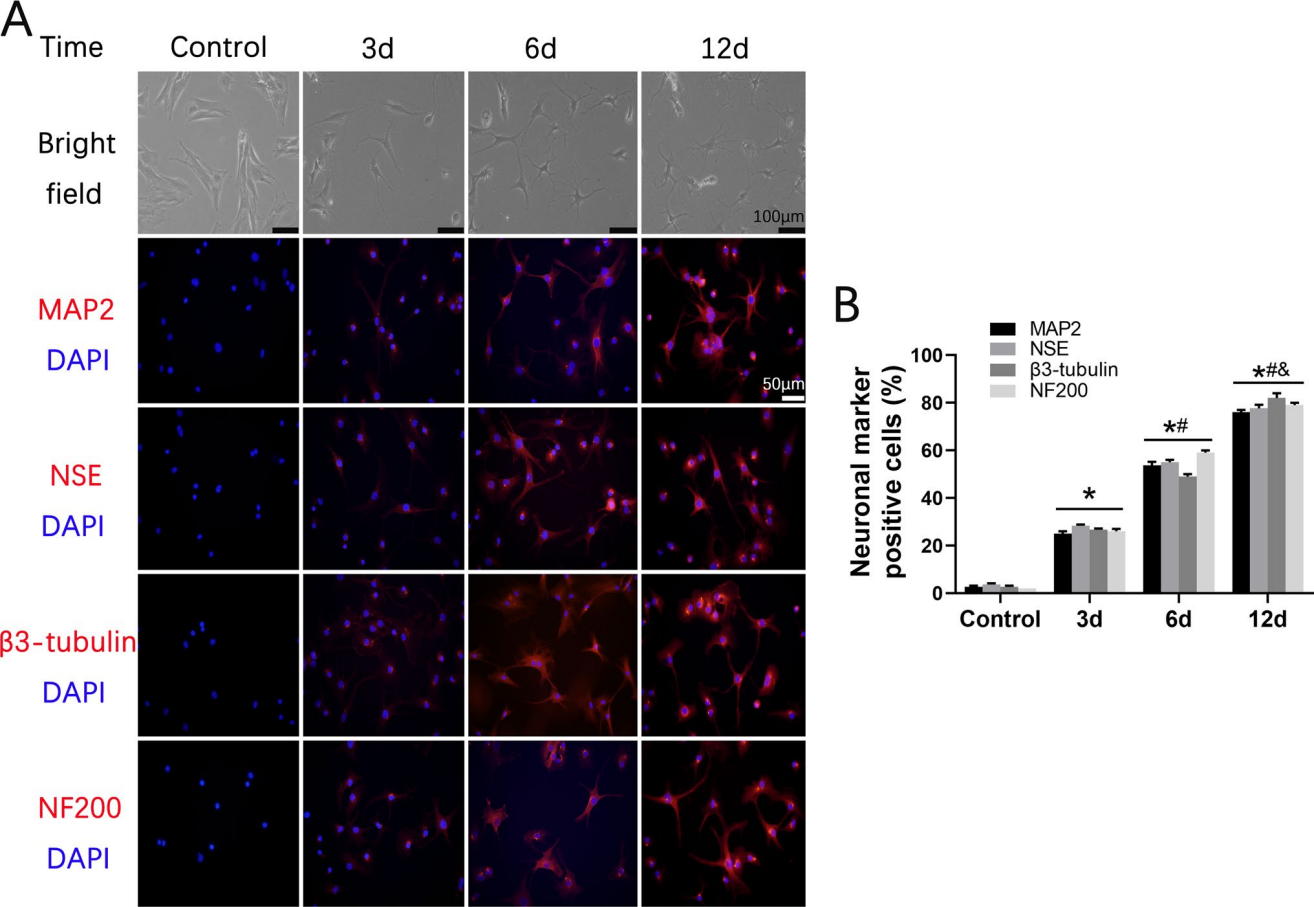
BMSCs were successfully performed neuronal differentiation. **A** Bright fields and immunofluorescent staining of BMSCs treated with neuronal differentiation induction medium for 3, 6, and 12 days, respectively, and undifferentiated induced BMSCs were used as control. **B** Percentage of neural- differentiated markers positive cells of BMSCs. Data are presented with mean ± SD; *P < 0.05 vs. the control group; ^#^*P* < 0.05 vs. the 3d group; ^&^*P* < 0.05 vs. the 6d group

To assess the efficacy of neuronal differentiation in BMSCs, a neuronal differentiation induction medium was administered to the BMSCs for durations of 3, 6, and 12 days. Undifferentiated-induced BMSCs were employed as the control group. Upon neuronal induction, bright field images demonstrated neurite-like outgrowth in the BMSCs. Furthermore, immunofluorescence analysis exhibited a significant increase in the presence of MAP2, NSE, β3-tubulin, and NF200 (neuronal markers) positive cells with prolonged induction time (Fig. 1A, B). These findings unequivocally demonstrate the successful induction of BMSCs into neuronal cells, whereby the efficiency of BMSCs’ neuronal differentiation gradually improved over time (3, 6, and 12 days).

### Notch1 Downregulation by miR-28-5p During BMSC Neuronal Differentiation In Vitro

**Fig. 2:**
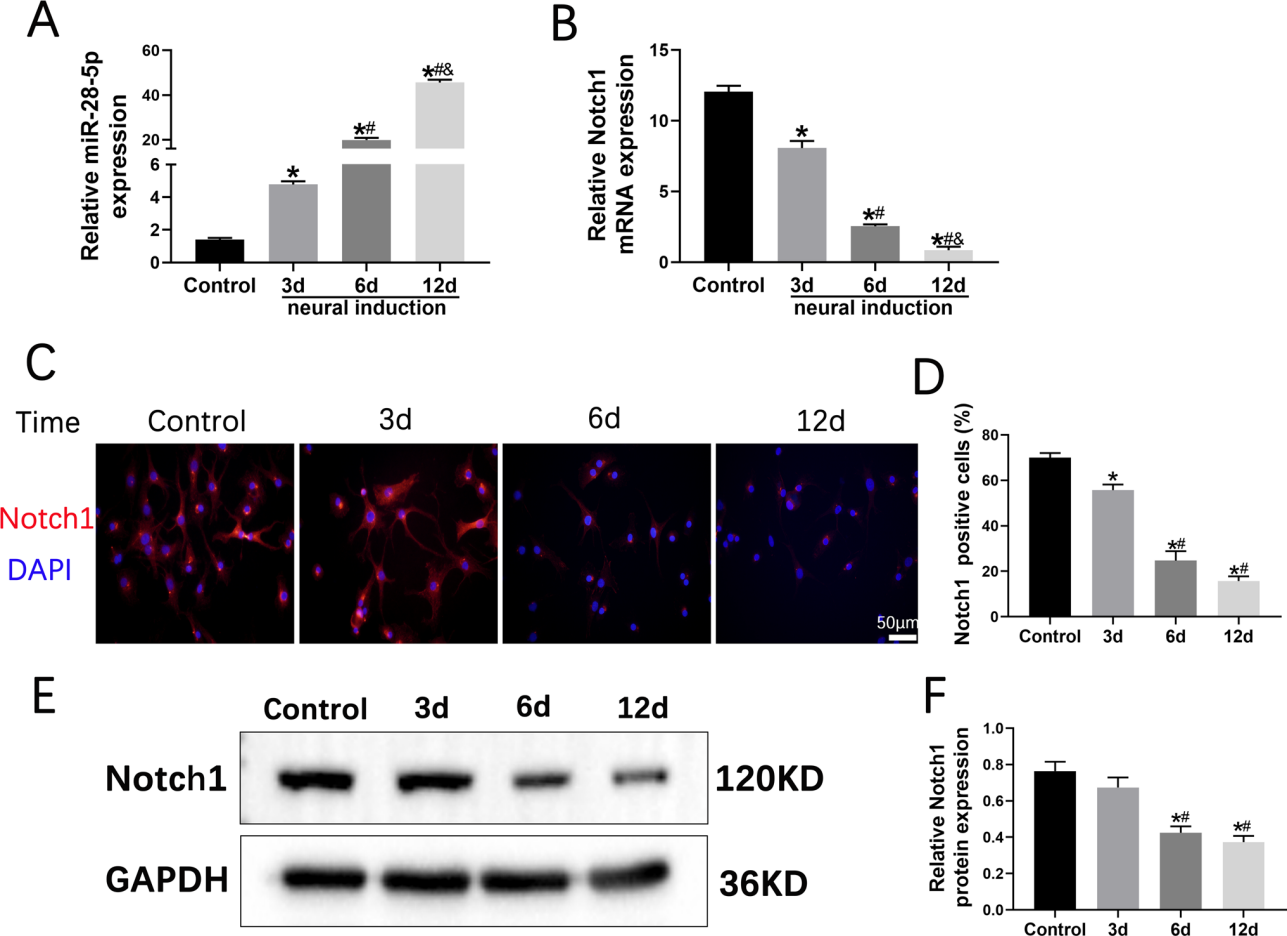
miR-28-5p negatively regulated Notch-1 during BMSCs neuronal differentiation in vitro**. A, B** qRT-PCR was performed to detect the mRNA expression of miR-28-5p and Notch1 during BMSCs neuronal differentiation for 3 days, 6 days, and 12 days, and undifferentiated induced BMSCs were used as control. **C, D** Immunofluorescent staining and relative neural- differentiated markers positive cells showing the protein expression of Notch-1 during BMSCs neuronal differentiation for 3 days, 6 days, and 12 days. **E, F** Western blot analysis and relative quantification showing the protein expression of Notch-1 during BMSCs neuronal differentiation for 3 days, 6 days, and 12 days. GAPDH was used as the internal control. Data are presented with mean ± SD; *P < 0.05 vs. the control group; ^#^*P* < 0.05 vs. the 3d group; ^&^*P* < 0.05 vs. the 6d group.

To assess the expression of miR-28-5p and Notch1 during neuronal differentiation of BMSCs, we subjected the BMSCs to a differentiation induction medium for 3, 6, and 12 days, as well as undifferentiated-induced BMSCs. Subsequently, qRT-PCR was employed to analyze the samples. The findings revealed a significant increase in miR-28-5p expression throughout the differentiation period (3, 6, and 12 days), while Notch-1 expression demonstrated a noticeable decrease (Fig. 2A, B). Notably, a consistent decline in Notch-1 expression was observed during BMSC neuronal differentiation. These trends were corroborated by immunofluorescence and Western blot analyses, which demonstrated similar effects on Notch-1 protein expression (Fig. 2C–F). Consequently, it can be inferred that miR-28-5p potentially exerts negative regulation on Notch-1 during in vitro neuronal differentiation of BMSCs.

### miR-28-5p May Directly Targets Notch-1

**Fig. 3:**
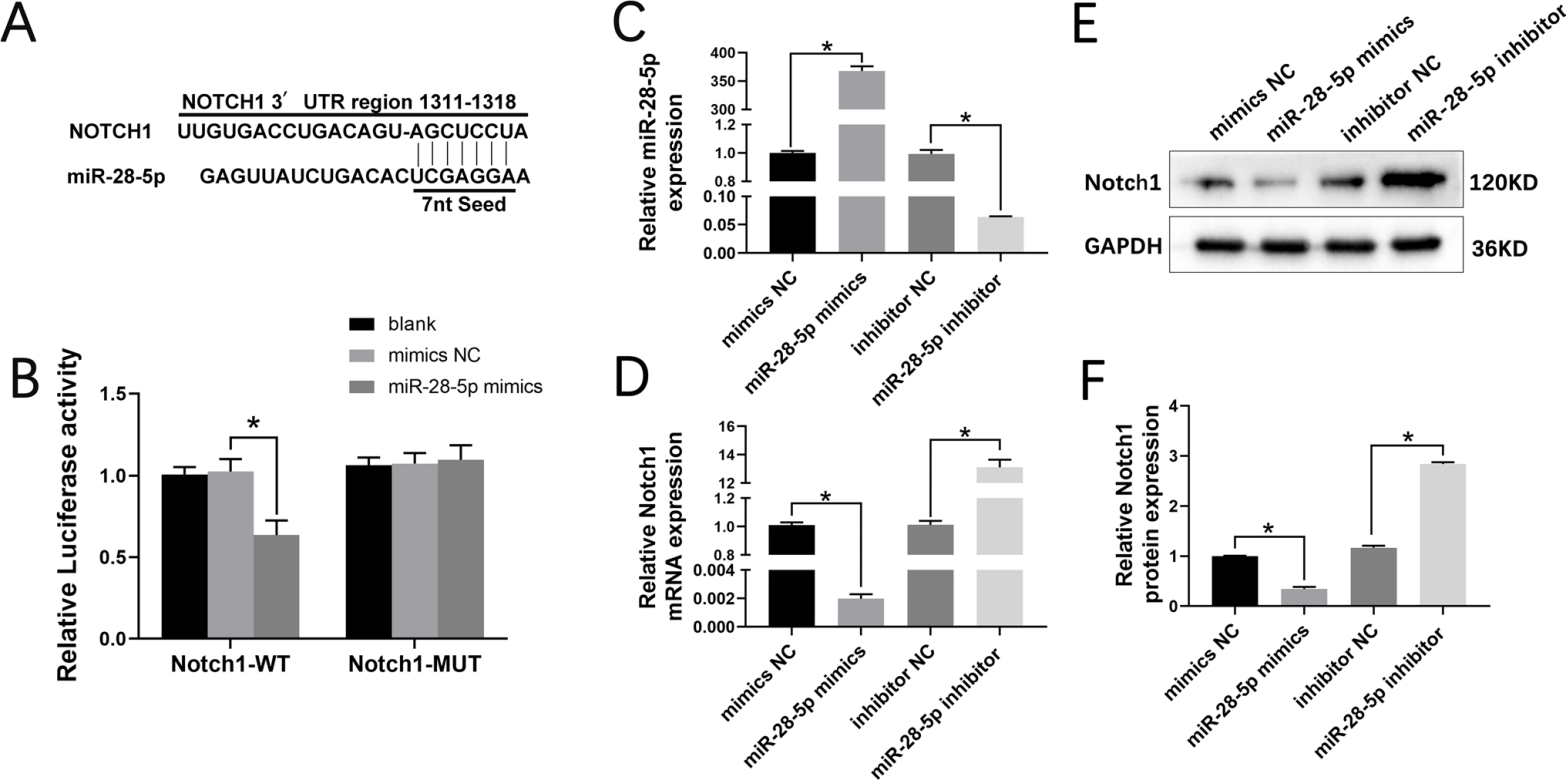
miR-28-5p directly targets Notch-1**. A** A putative miR-28-5p-binding site existed in the Notch1 mRNA. **B** The relative luciferase activity between cells was compared after co-transfection of Notch-1-WT/ Notch-1-MUT vectors with miR-28-5p mimics and negative control. **C, D** qRT-PCR was performed to detect the mRNA expression of miR-28-5p and Notch-1. **E, F** Western blot analysis and relative quantification showing the protein expression of Notch1 in BMSCs. Data are presented with mean ± SD, *P < 0.05.

The TargetScan software identified Notch1 as the target gene of miR-28-5p (Fig. 3A). To investigate whether miR-28-5p regulates Notch-1 expression, we generated WT and MUTNotch1 3′-UTR fragments, which were subsequently cloned downstream of the dual-luciferase reporter gene. Luciferase activity assays performed in 293 T cells showed that the miR-28-5p mimic had no effect on the luciferase activity of Notch1-MUT cells but significantly inhibited the activity of Notch1-WT cells (*p* < 0.05; Fig. 3B). These results provide evidence that miR-28-5p may directly targets Notch-1.

To further explore the regulatory relationship between miR-28-5p and Notch-1, we transfected BMSCs with miR-28-5p mimics, inhibitors, and a negative control. The qRT-PCR analysis revealed a significant decrease in Notch1 mRNA levels in miR-28-5p mimic-treated BMSCs compared to the control group. Conversely, the miR-28-5p inhibitor significantly enhanced Notch-1 expression (Fig. 3C, D). Western blotting analysis confirmed similar effects on Notch-1 protein expression (Fig. 3E, F). Therefore, it can be concluded that miR-28-5p downregulates Notch-1 expression.

### miR-28-5p Facilitated BMSC Neuronal Differentiation In Vitro

Various microRNAs (miRNAs) have been shown to promote the self-renewal and differentiation of stem cells by downregulating specific target genes. Consequently, we aimed to investigate the impact of miR-28-5p on neuronal differentiation of BMSCs in vitro. Thus, we transfected BMSCs with either miR-28-5p mimics or inhibitors and induced neuronal differentiation over a period of 12 days. Subsequently, we evaluated the neuronal specificity by examining the expression of neuronal markers (MAP2, NSE, β3-tubulin, and NF200) through immunofluorescent staining.

**Fig. 4:**
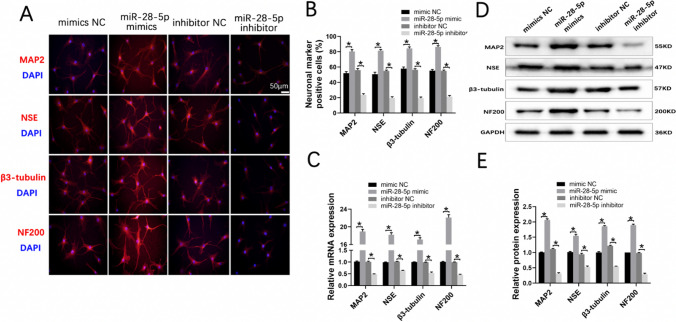
miR-28-5p facilitated BMSCs neuronal differentiation in vitro**. A, B** Immunofluorescence showing the protein expression of the neuronal markers including MAP2, NSE, β3-tubulin, and NF200 in differentiated BMSCs transfected with miR-28-5p mimics, inhibitor, and their negative control at 12 days. **C** qRT-PCR showing the mRNA expression of the neuronal markers including MAP2, NSE, β3-tubulin, and NF200 in differentiated BMSCs at 12 days. **D, E** Western blot analysis showing the protein expression of the neuronal markers in differentiated BMSCs at 12 days. Data were presented with mean ± SD,*P < 0.05 vs. control group.

The results of immunofluorescent staining demonstrated a significant increase in the number of MAP2, NSE, β3-tubulin, and NF200 positive cells in the mimics group compared to the mimics NC group. Conversely, the inhibitor group displayed a considerably lower count of neuronal marker positive cells compared to the inhibitor NC group (Fig. 4A, B). Consistent trends in the expression of neuronal markers were observed at the mRNA level (Fig. 4C). Furthermore, Western blot analysis revealed a significant upregulation of MAP2, NSE, β3-tubulin, and NF200 protein levels in BMSCs treated with miR-28-5p mimics, while the inhibition of miR-28-5p led to the opposite outcome (Fig. 4D, E). Collectively, these findings strongly indicate that miR-28-5p promotes neuronal differentiation of BMSCs in vitro over a 12-day duration.

### Transplantation of Lentivirus-Mediated miR-28-5p-Overexpressed BMSCs Contributed to Tissue Repairing and Functional Recovery of SCI Rats

Next, we examined the effects of miR-28-5p in vivo. Lentivirus-mediated miR-28-5p-overexpressed BMSCs and negative control were constructed and then transplanted into rats with TSCI via tail vein injection on the first day after surgery. To assess tissue repair, functional recovery, and nerve cell regeneration, we employed various evaluation methods including behavioral observation images, footprint tests, BBB scores, HE staining, Nissl staining, and immunofluorescent staining.

**Fig. 5:**
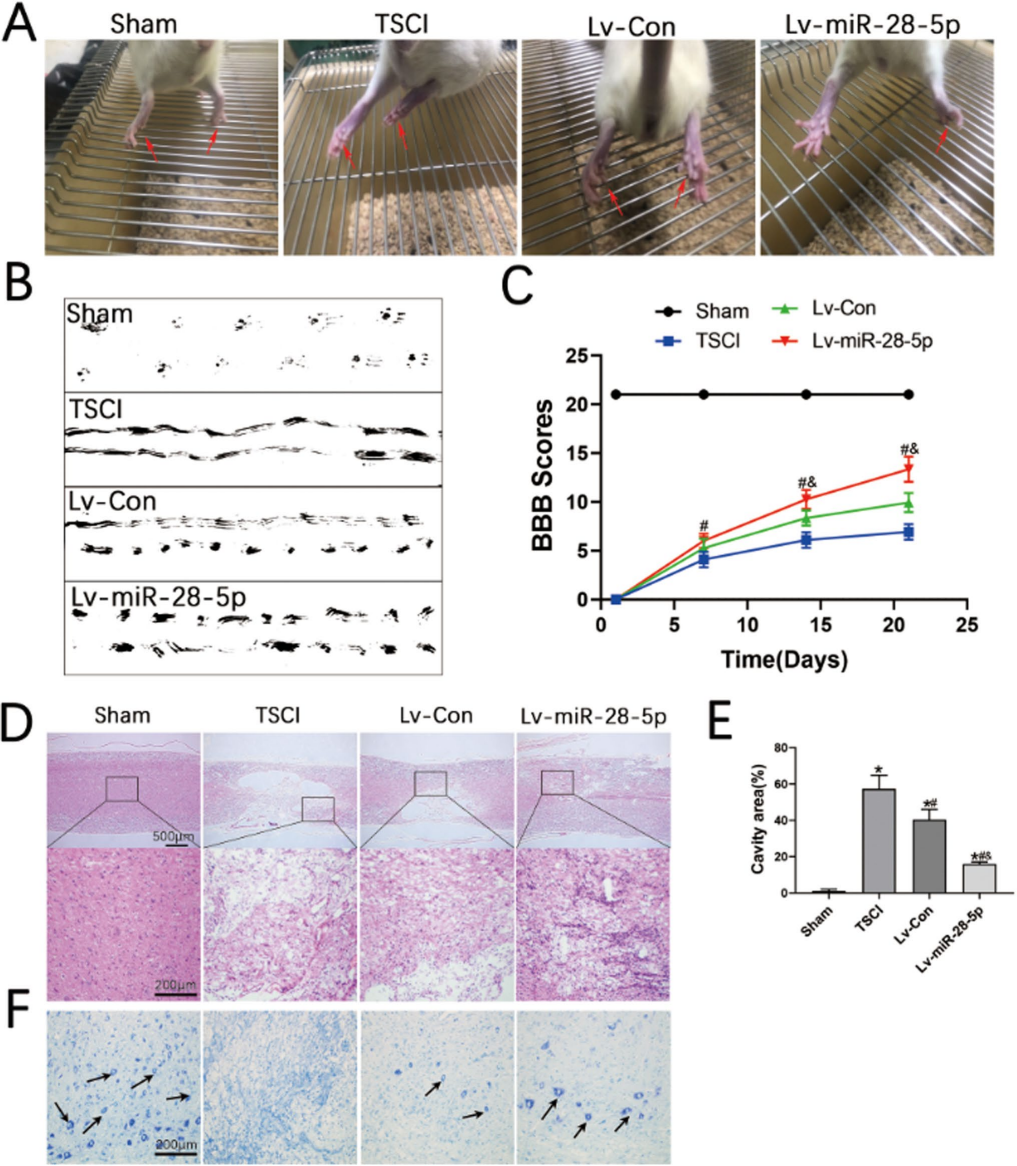
Transplantation of lentivirus-mediated miR-28-5p-overexpressed BMSCs contributed to tissue repairing and functional recovery of SCI rats**. A** Behavioral observation images showing the hind limbs movement characteristics of rats. **B** Footprint test at 21 days after BMSCs transplantation. **C** The Basso, Beattie, and Bresnahan (BBB) locomotor scores. **D, E** HE staining in longitudinal section and relative quantification of the cavity area showing the histological morphology of the different groups at 21 days after BMSCs transplantation. **F** Nissl staining at the lesion area were performed to evaluate the survival neurons of the different groups at 21 days after BMSCs transplantation. Data were presented with mean ± SD; *P < 0.05 vs Sham group; ^#^*P* < 0.05 vs. SCI group; ^&^*P* < 0.05 vs. Lv-Con group

In the Sham group, rats exhibited strong hindlimbs that allowed for easy grasping and walking (Fig. 5A). However, the TSCI group displayed weak hindlimbs that could neither grasp nor walk. Encouragingly, rats treated with miR-28-5p-overexpressed BMSCs showed restoration of hindlimb strength and slow improvement in grasping and walking ability. To analyze the effect of behavioral function repair across different groups, we conducted footprint tests and assessed BBB scores to evaluate hindlimb locomotor activity after SCI. In the footprint tests, SCI rats dragged both hindlimbs and were unable to walk, while those treated with miR-28-5p-overexpressed BMSCs presented relatively consistent footprints in both hindlimbs and regained some strength 21 days after transplantation. Furthermore, rats treated with miR-28-5p-overexpressed BMSCs exhibited an advantage over the NCs in the footprint test (Fig. 5B). Except for the Sham group, all rats had zero hindlimb movements after surgery. Compared to the TSCI group, the Lv-miR-28-5p group demonstrated increased BBB scores on days 7, 14, and 21. Specifically, rats treated with miR-28-5p-overexpressed BMSCs had higher BBB scores than the Lv-Con group on days 14 and 21 (Fig. 5C).

To observe the histological morphology of the rat’s spinal cords, we performed HE staining 21 days after BMSC transplantation. Compared to normal rats, significant deformities and cavities were detected at the site of spinal cord injury in SCI rats (Fig. 5D, E). However, in rats treated with miR-28-5p-overexpressed BMSCs for 21 days, the lesion cavity area significantly decreased, and there was a reduction in tissue damage compared to the TSCI and Lv-Con groups.

Furthermore, Nissl staining was used to assess neuron survival 21 days post-BMSC transplantation. Notably, the neuron survival at the lesion area in rats treated with miR-28-5p-overexpressed BMSCs significantly increased compared to both the TSCI and Lv-Con groups (Fig. 5F). Overall, these results suggest that the transplantation of lentivirus-mediated miR-28-5p-overexpressed BMSCs plays a role in promoting tissue repair and functional recovery in vivo.

### Transplantation of Lentivirus-Mediated miR-28-5p-Overexpressed BMSCs Facilitated Neuronal Regeneration of SCI Rats by Notch-1 Targeting

**Fig. 6:**
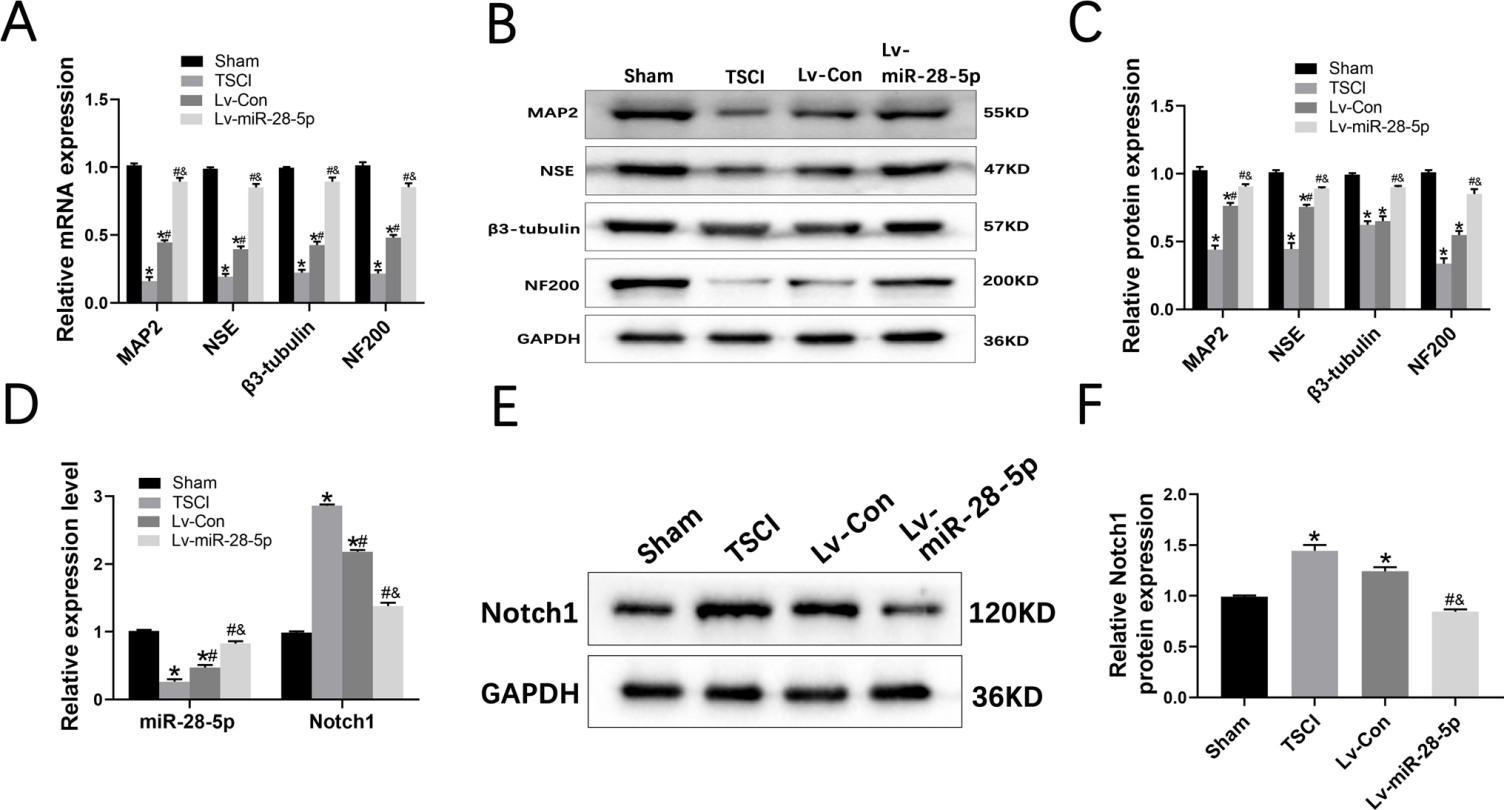
Transplantation of lentivirus-mediated miR-28-5p-overexpressed BMSCs facilitated neuronal regeneration of SCI rats by targeting Notch1**. A** qRT-PCR showing the mRNA expression of the neuronal markers including MAP2, NSE, β3-tubulin, and NF200 of the different groups at 21 days after BMSCs transplantation. **B, C** Western blot analysis showing the protein expression of the neuronal markers of the different groups at 21 days after BMSCs transplantation. **D** qRT-PCR showing the mRNA expression of the miR-28-5p and Notch1 of the different groups at 7 days after BMSCs transplantation. **E, F** Western blot analysis and relative quantification showing the protein expression of the Notch1 of the different groups at 7 days after BMSCs transplantation. Data were presented with mean ± SD; *P < 0.05 compared with the Sham group; ^#^*P* < 0.05 vs. SCI group; ^&^*P* < 0.05 vs. Lv-Con group

The present study employed qRT-PCR and Western blot techniques to investigate the in vivo neuronal regeneration effect of miR-28-5p-overexpressed BMSCs. The qRT-PCR analysis revealed a significant increase in the expression of neuronal markers (MAP2, NSE, β3-tubulin, and NF200) in miR-28-5p-overexpressed BMSCs compared to the SCI group 21 days post-transplantation. Furthermore, the Lv-miR-28-5p group exhibited significantly higher expression levels of these neuronal markers compared to the Lv-Con group (Fig. 6A). A similar pattern was observed at the protein level (Fig. 6B, C), indicating consistent trends in neuronal marker levels.

Additionally, the qRT-PCR results demonstrated a significant decrease in miR-28-5p expression in the TSCI group, whereas rats treated with miR-28-5p-overexpressed BMSCs showed a significant increase in miR-28-5p levels 7 days after transplantation. Conversely, Notch-1 mRNA levels increased in the TSCI group; however, miR-28-5p overexpression significantly reversed this trend (Fig. 6D). Consistent with these findings, Western blot analysis exhibited comparable effects on Notch-1 at the protein level (Fig. 6E, F). Taken together, these results suggest that the transplantation of lentivirus-mediated miR-28-5p-overexpressed BMSCs promotes neuronal regeneration in SCI rats through targeted inhibition of Notch-1.

### Transplantation of Lentivirus-Mediated miR-28-5p-Overexpressed BMSCs Promoted Axonal Regeneration of SCI Rats

**Fig. 7:**
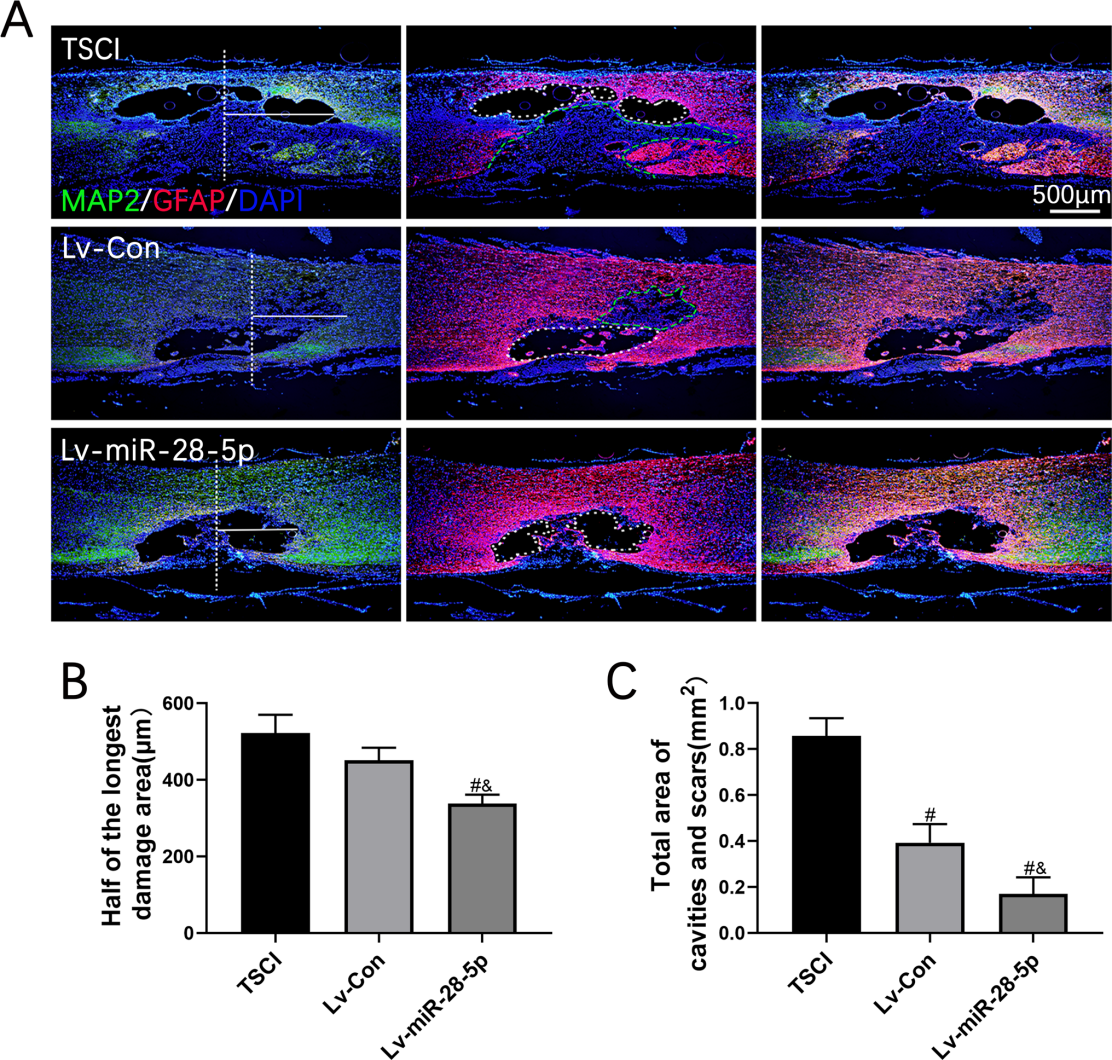
Transplantation of lentivirus-mediated miR-28-5p-overexpressed BMSCs decreased the tissue and neurons damage of SCI rats**. A** Co-immunofluorescent staining showed GFAP (red) and MAP2 (green) at 21 days after BMSCs transplantation. **B** Quantification of the maximum radius of injured areas of spinal cord from MAP2 immunoflurescent staining. **C** Quantification of the total area of cavities and necrotic of injured areas from GFAP immunoflurescent staining. Data were presented with mean ± SD; ^#^*P* < 0.05 vs. SCI group; ^&^*P* < 0.05 vs. Lv-Con group

We further employed immunofluorescent staining to investigate the effects of glial scar and axonal regeneration on functional recovery in SCI rats treated with miR-28-5p-overexpressed BMSCs. At 21 days post-BMSC transplantation, we evaluated tissue continuity at the lesion center using MAP2 (green) and GFAP (red). We utilized immunofluorescent staining of MAP2 to measure the maximum radius of cavities and necrotic areas within the lesion center. Comparatively, SCI rats treated with miR-28-5p-overexpressed BMSCs exhibited a significant reduction in the maximal radius of the lesion area compared to TSCI and Lv-Con rats. Furthermore, GFAP-positive astrocytes were activated and surrounded the lesion site, playing a reparative role accompanied by the formation of cavities and necrotic areas. In the TSCI group, cavities and necrotic areas surrounded by astrocytes were observed, but no MAP-positive axons were found at the lesion site (Fig. 7A–C).

The total area of cavities and necrosis in the Lv-miR-28-5p group was significantly smaller than that in the TSCI and Lv-Con groups. Additionally, SCI rats treated with miR-28-5p-overexpressed BMSCs showed an abundance of MAP-positive axons extending into the lesion sites, partially bridging the cavities and glial scar areas, thereby promoting tissue continuity. These findings indicate that transplantation of lentivirus-mediated miR-28-5p-overexpressed BMSCs effectively reduced tissue damage in SCI rats.

The formation of glial scars poses a major physiological barrier to axon regeneration following SCI. Therefore, we performed double immunofluorescent staining of GFAP and GAP43 to investigate glial scar formation and axonal regeneration in the injured area. GAP43 is closely linked to neuronal regeneration and can regulate axonal growth and the formation of new connections.

**Fig. 8:**
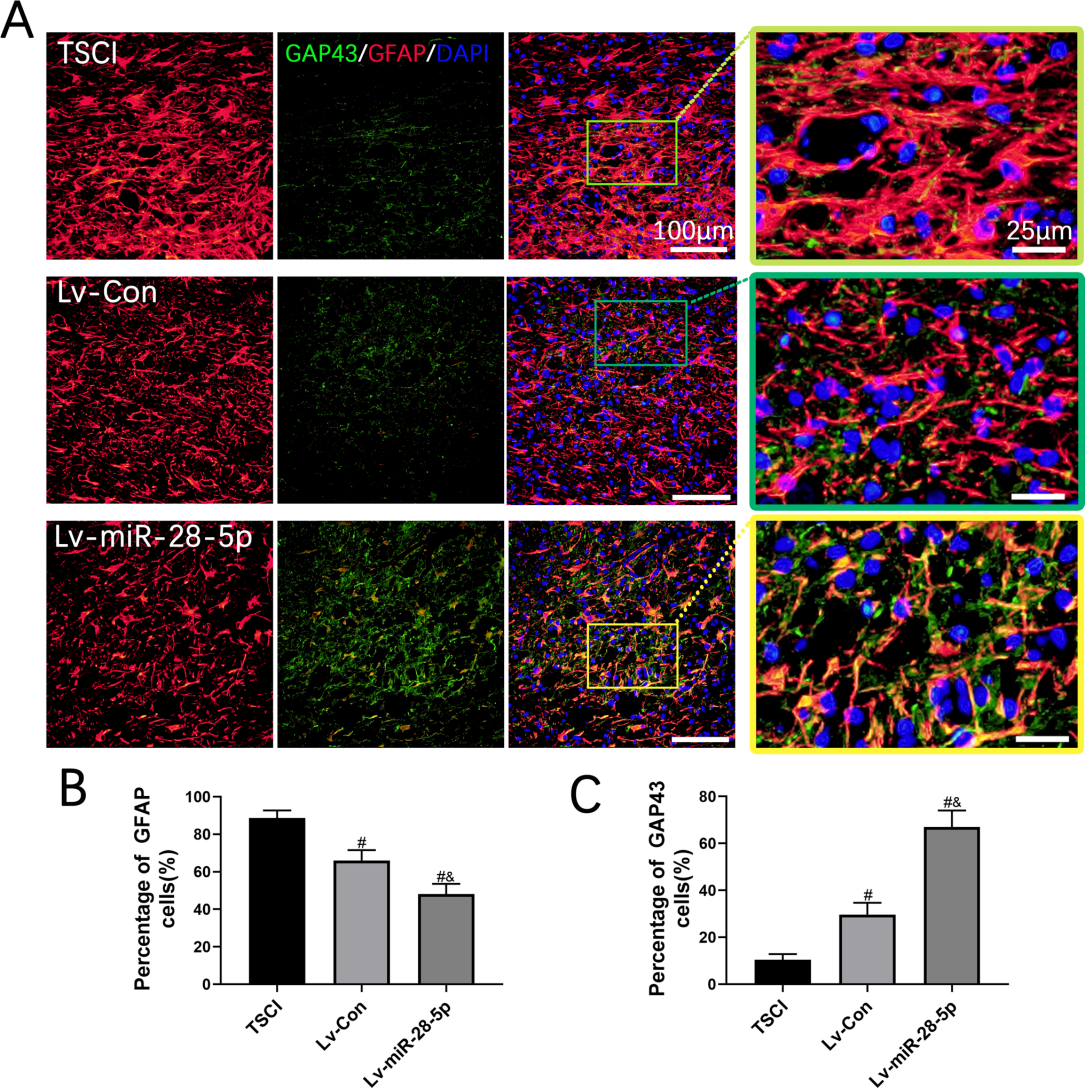
Transplantation of lentivirus-mediated miR-28-5p-overexpressed BMSCs decreased the formation of glial scar and promoted axonal regeneration of SCI rats**. A** Co-immunofluorescent staining showed the glial scar (GFAP, red) and the axonal regeneration (GAP43, green) at 21 days after BMSC transplantation. **B** Quantification of GFAP immunoflurescent staining. **C** Quantification of GAP43 immunoflurescent staining. Data were presented with mean ± SD; ^#^*P* < 0.05 vs. SCI group; ^&^*P* < 0.05 vs. Lv-Con group

In the TSCI and Lv-Con groups, we observed distinct GFAP-positive glial scars and few GAP43-positive axons. Conversely, SCI rats treated with miR-28-5p-overexpressed BMSCs exhibited a significant decrease in GFAP-positive glial scar formation accompanied by a considerable increase in GAP43-positive axons (Fig. 8A–C). These results collectively demonstrate that transplantation of lentivirus-mediated miR-28-5p-overexpressed BMSCs not only reduces glial scar formation but also promotes axonal regeneration in SCI rats.

### Transplantation of Lentivirus-Mediated miR-28-5p-Overexpressed BMSCs Contributed to Axonal Remyelination of SCI Rats

**Fig. 9:**
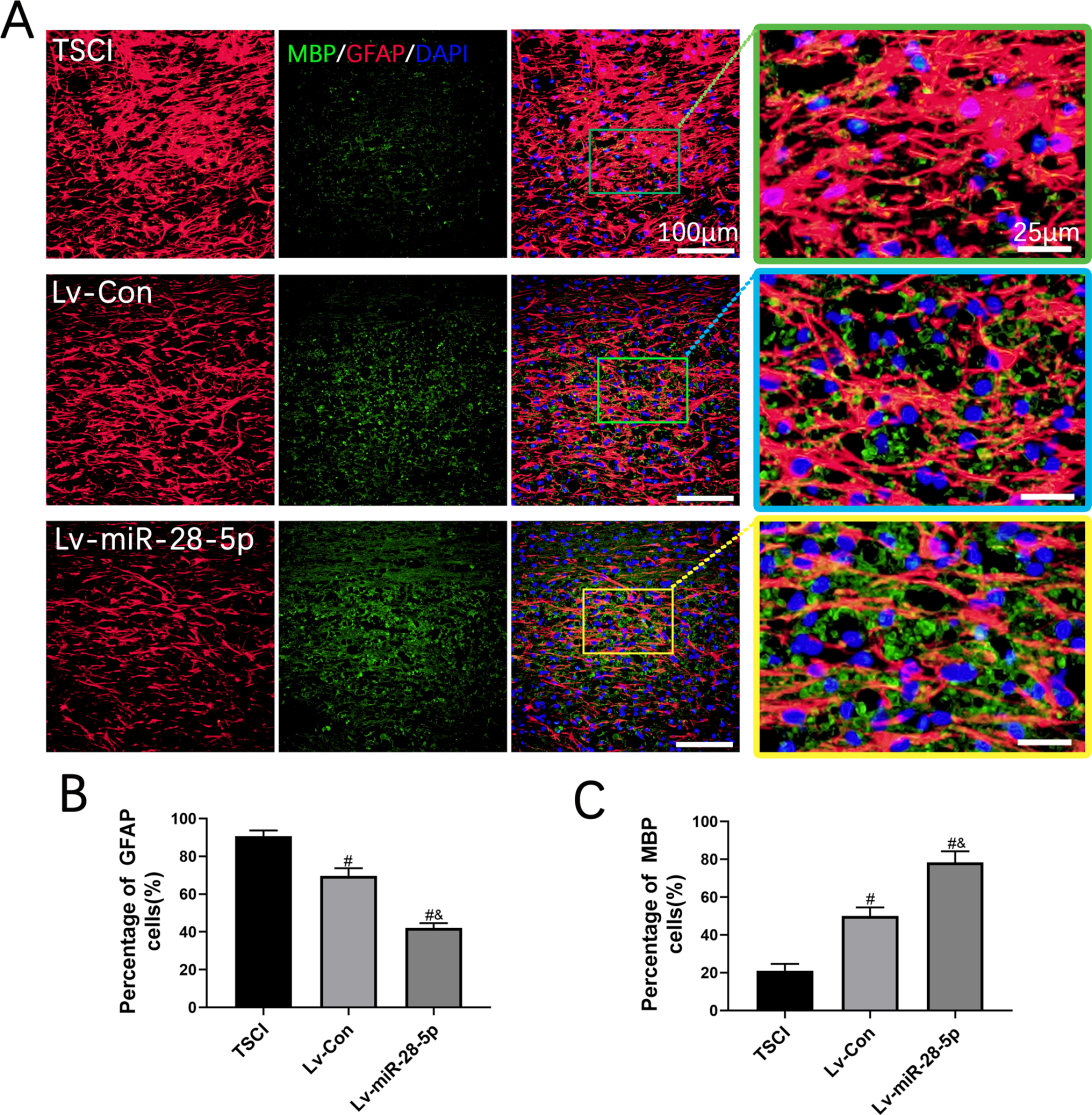
Transplantation of lentivirus-mediated miR-28-5p-overexpressed BMSCs contributed to axonal remyelination of SCI rats**. A** Co-immunofluorescent staining showed the glial scar (GFAP, red) and the axonal remyelination (MBP, green) at 21 days after BMSCs transplantation. **B** Quantification of GFAP immunoflurescent staining. **C** Quantification of MBP immunoflurescent staining. Data were presented with mean ± SD; ^#^*P* < 0.05 vs. SCI group; ^&^*P* < 0.05 vs. Lv-Con group

Axonal myelination plays a crucial role in the maintenance of neurological functions. The detection of active remyelination relies heavily on the MBP, a significant component of myelin. Therefore, to examine the impact of miR-28-5p on remyelination, we employed double immunofluorescence staining for MBP and GFAP at 21 days post-BMSC transplantation. At the lesion site in the SCI group, MBP expression was found to be low. However, in the Lv-miR-28-5p group, MBP levels exhibited a significant increase compared to both the TSCI and Lv-Con groups. Conversely, GFAP levels demonstrated a significant decrease in the Lv-miR-28-5p group when compared to the TSCI and Lv-Con groups (Fig. 9A–C). These findings indicate that the transplantation of lentivirus-mediated miR-28-5p-overexpressing BMSCs promoted axonal remyelination in rats with SCI.

## Discussion

TSCI is characterized by the necrosis or apoptosis of neurons and glial cells, demyelination, axon degeneration, and loss of neural circuits, resulting in temporary or permanent loss of motor and sensory abilities, and even paralysis [24]. Stem cells have shown potential for reconstructing the nervous system due to their ability to proliferate, migrate, and differentiate into neuron-like cells [25–27]. Previous studies have demonstrated that stem cell transplantation can increase spinal nerve cells and reduce the formation of keratinous scars and cavities in animal models of SCI, which has become a recent focal point in medical research [28, 29]. Among various types of stem cells, BMSCs are favored due to their potent differentiation capacity, wide availability, simplicity of acquisition, and low transplant reaction [30, 31]. However, the limited ability of BMSCs to direct neuronal differentiation and the low survival rate of differentiated neural cells severely restrict their clinical application. Therefore, enhancing the differentiation ability of BMSCs and the viability of differentiated cells is crucial for improving the efficacy of stem cell therapy.

Prior studies have indicated the close relationship between certain miRNAs and biological processes such as inflammation, regeneration, apoptosis, and demyelination, making them essential for mammalian neuronal differentiation [13, 14]. Among them, miR-28-5p has been found to play a significant role in the progression of different cancers, neuropathic pain, and neurodegeneration [15–17, 32, 33]. In models of chronic sciatic nerve injury and spinal cord ischemia–reperfusion injury, miR-28-5p was significantly downregulated, suggesting its involvement in neural injury and repair [16, 17]. Additionally, miR-28-5p has been associated with the proliferation of various stem cells [34–36]. Therefore, we investigated the role of miR-28-5p in the differentiation process of BMSCs and analyzed its mechanism. Ultimately, we discovered that miR-28-5p downregulated Notch-1 and promoted the differentiation of BMSC neurons, thereby promoting the recovery of damaged spinal cord tissues and functions, axon regeneration, and remyelination.

Initially, we determined the increase in miR-28-5p expression during BMSC neuronal differentiation. Subsequently, we transfected BMSCs with miR-28-5p mimics or inhibitors and induced their differentiation. The results showed a significant increase in MAP2, NSE, β3-tubulin, and NF200-positive cells (neuronal markers) compared to the NC group. Furthermore, miR-28-5p inhibitors reversed these results. These findings demonstrated that miR-28-5p may regulate BMSC neuronal differentiation, although the specific regulatory mechanism remains unclear.

Target gene prediction suggested that Notch-1 might be the target gene of miR-28-5p. Notch-1 is the most important subtype of Notch receptors and is a transmembrane protein that regulates cell proliferation, differentiation, and apoptosis through signal transmission with adjacent cells [37]. In the central nervous system (CNS), Notch signaling governs the growth, development, and activation of nerve cells [38]. As a regulator of NSCs, Notch signaling affects their proliferation, differentiation, and the maturation of the nervous system, including neuronal differentiation [39–41]. Notch signaling has also been shown to influence neuron-like differentiation in other stem cells [42]. For instance, wogonin inhibits Notch1 signaling and promotes retinal neuron-like differentiation of BMSCs [43]. In our study, bioinformatics and dual-luciferase analyses, qRT-PCR, and Western blot validated the targeting relationship between miR-28-5p and Notch-1. We observed a gradual decrease in Notch-1 expression during BMSC neuronal differentiation, contrasting the trend in miR-28-5p expression. These results indicated that miR-28-5p facilitated BMSC neuronal differentiation by targeting Notch-1.

Despite the potential of cell transplantation, its success depends on the survival of transplanted cells [6, 7]. Unfortunately, a large proportion of BMSCs undergo apoptosis or differentiate into other functional cells after transplantation, resulting in suboptimal therapeutic outcomes. Only a small proportion of them successfully differentiate into neurons, further limiting the efficacy of the treatment [44, 45]. Therefore, promoting the directional ability of BMSC neuronal differentiation and improving cell survival rate after neuronal differentiation are crucial.

**Fig. 10:**
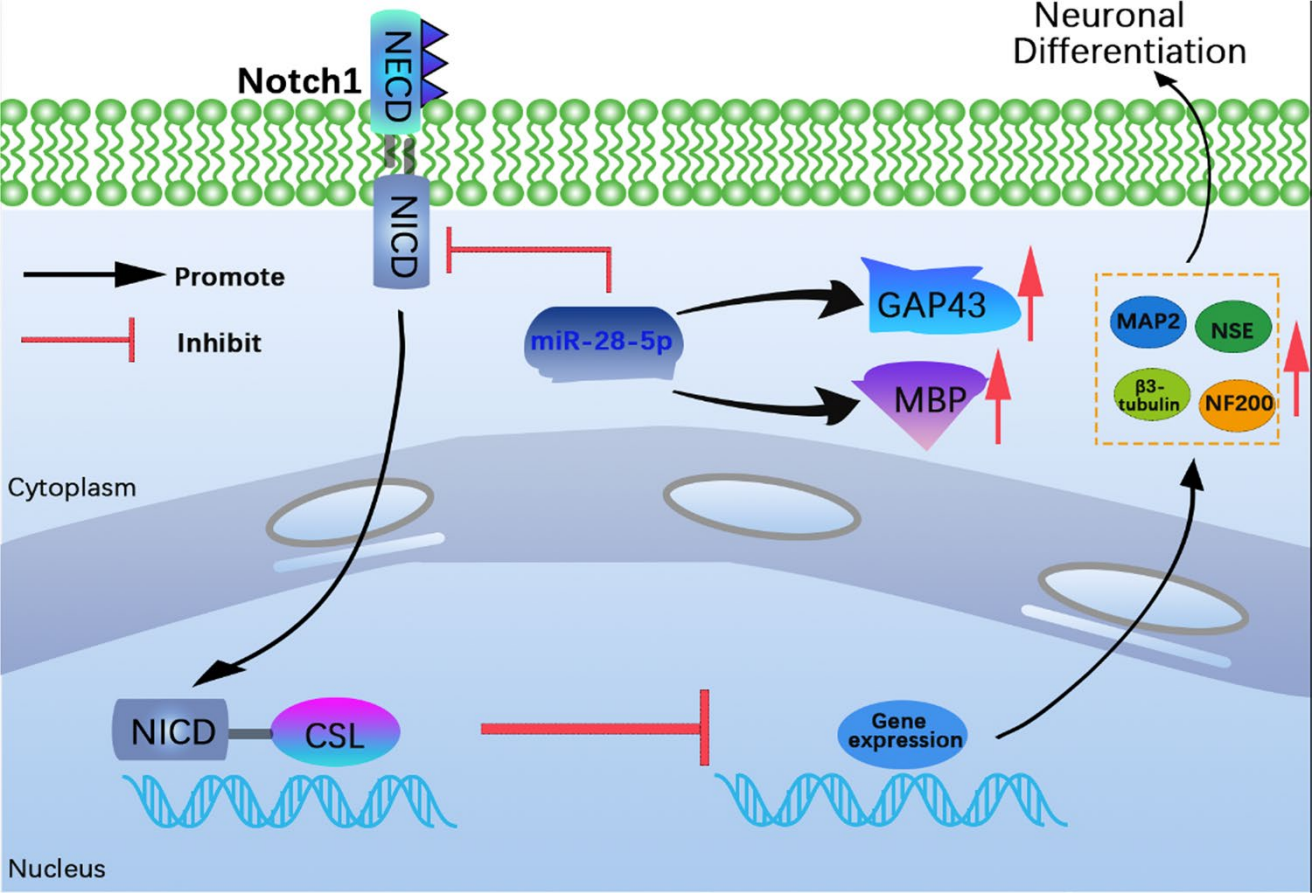
A schematic diagram

To investigate whether miR-28-5p could promote directed differentiation of BMSCs into neurons in vivo and improve the efficacy of treating SCI, we constructed lentivirus-mediated miR-28-5p-overexpressed BMSCs and grafted them into SCI rats. The results revealed significant improvement in behavioral observation images, footprint tests, BBB scores, and histological morphology in SCI rats treated with miR-28-5p-overexpressed BMSCs. Additionally, qRT-PCR and Western blot demonstrated that BMSCs facilitated neuronal regeneration in SCI rats. Moreover, a decrease in Notch-1-related mRNA and protein levels was observed in these rats. These findings indicated that miR-28-5p overexpression might facilitate neuronal regeneration in SCI rats by targeting Notch-1 (Fig. 10).

Furthermore, we examined pathological tissues to evaluate the efficacy of miR-28-5p in vivo. At the lesion site, primary mechanical injury typically involves hemorrhage, ischemia, and massive local neuronal death. Edema and inflammation further develop, leading to the expansion of necrotic areas and cavities surrounded by fibrotic scars, ultimately causing severe secondary injury and neurological dysfunction [46]. In this study, evaluation of the lesion center 21 days after BMSC transplantation revealed a significant reduction in the maximum radius of the injury area, total cavity area, and necrotic area in SCI rats treated with miR-28-5p-expressing BMSCs, consistent with previous reports [47, 48]. These results indicate that miR-28-5p overexpression could decrease tissue damage in SCI rats.

Glial scar formation acts as a physical and chemical barrier inhibiting axonal regeneration and controlling the progression of secondary injury [46, 49]. Thus, reducing the area of glial scars and cavities to promote axonal regeneration and remyelination is crucial for functional recovery after SCI. Our study confirmed the effects of miR-28-5p on glial scar formation in SCI rats. Double GFAP and GAP43 immunofluorescent staining revealed a significant increase in GFAP expression 21 days after BMSC transplantation, indicating the formation of glial scars with GFAP-positive astrocytes at the lesion sites. Cavities and necrotic areas surrounded by astrocytes were also observed in the SCI region. However, GFAP expression decreased in SCI rats treated with miR-28-5p-overexpressed BMSC transplantation. These findings are consistent with previous reports [50] and suggest that lentivirus-mediated miR-28-5p-overexpressed BMSC transplantation reduced glial scar formation and promoted axonal regeneration in SCI rats, bridging the gap at lesion sites and restoring tissue continuity.

Myelin plays a critical role in maintaining axon integrity and neurological functions [51]. After SCI, secondary injury causes edema, inflammation, oligodendrocyte necrosis, and apoptosis, leading to axonal demyelination. The degree of remyelination directly affects SCI repair. Spontaneous remyelination accounts for only a small proportion, and persistent demyelination eventually leads to irreversible neurological loss [52]. Therefore, promoting remyelination is vital for axonal regeneration. We performed co-immunofluorescence staining of MBP and GFAP to assess remyelination 21 days after BMSC transplantation. The levels of MBP in the miR-28-5p overexpression group significantly increased compared to controls, consistent with previous reports [53, 54]. Overall, these results demonstrate that miR-28-5p overexpression contributes to the axonal remyelination of SCI rats.

There are some limitations in our current study. Firstly, the detailed mechanisms underlying miR-28-5p-mediated neural differentiation and regeneration remain unclear; we have only indicated that miR-28-5p may directly target Notch-1 to promote neuronal differentiation. Secondly, we did not use gene knockout or transgenic mice in vivo, which may warrant further research in the future.

## Conclusion

In this study, our present findings may provide evidence that miR-28-5p may directly target Notch1, thereby facilitating the in vitro neuronal differentiation of BMSCs in vitro*.* Moreover, the transplantation of lentivirus-mediated miR-28-5p-overexpressed BMSCs into rats with SCI effectively inhibits Notch-1 and contributes to tissue repair, functional recovery, axonal regeneration, and axonal remyelination. Taken together, these results may suggest the potential of miR-28-5p-modified BMSCs as a therapeutic target for promoting behavioral and neurological recovery in SCI rats.

## Data Availability

The data used to support the findings of this study are available from the corresponding author upon request.

## Abbreviations

SCI: Spinal cord injury
BMSCs: Bone marrow mesenchymal stem cells
SD: Sprague-Dawley
PBS: Phosphate-buffered saline
FBS: Fetal bovine serum
BDNF: Brain-derived neurotrophic factor
EGF: Epidermal growth factor
bFGF: Basic fibroblast growth factor
WT: Wild type
MUT: Mutant
NC: Negative control
SPF: Specific pathogen-free
BBB: Basso, Beattie, and Bresnahan
PFA: Paraformaldehyde
HE: Hematoxylin-eosin
RT: Room temperature
MAP2: microtubule-associated protein 2
NSE: Neuron-specific enolase
NF-200: Neurofilament 200
RIPA: Radio immunoprecipitation assay
BCA: Bicinchoninic acid
PVDF: Polyvinylidene difluoride
TBST: Tris buffer solution
SDs: Standard deviations
MBP: Myelin basic protein
CNS: Central nervous system
